# X-Linked CGD Chorioretinitis in Two Young Girls

**DOI:** 10.3390/biomedicines13020323

**Published:** 2025-01-31

**Authors:** Johnathan Abraham Bailey, Maximilian Daechul Kong, Chanakarn Piamjitchol, Baichun Hou, Abdhel Exinor, Antara Nayak, Noah Heaps, Aykut Demirkol, Stephen H. Tsang

**Affiliations:** 1 Jonas Children’s Vision Care and Bernard & Shirlee Brown Glaucoma Laboratory, Institute of Human Nutrition, Columbia Stem Cell Initiative, New York, NY 10032, USA; jab2477@cumc.columbia.edu (J.A.B.); mk5010@cumc.columbia.edu (M.D.K.); ae2647@columbia.edu (A.E.); antara.nayak@lps-students.org (A.N.); nmh2153@columbia.edu (N.H.); ad3871@cumc.columbia.edu (A.D.); 2 Department of Ophthalmology, Bumrungrad International Hospital, Bangkok 10110, Thailand; mindchanakarn@gmail.com; 3 Edward S. Harkness Eye Institute, Columbia University Irving Medical Center, New York-Presbyterian Hospital, New York, NY 10032, USA

**Keywords:** chronic granulomatous disease, chorioretinitis, retina, x-linked inheritance, lyonization, full-field electroretinogram

## Abstract

Background: Chronic granulomatous disease (CGD) is a rare genetic disorder that causes primary immunodeficiency. In addition to increasing infection susceptibility in various bodily systems, several ocular manifestations have been described in males. This condition is well described in males, due to its X-linked recessive inheritance. However, here we present, to our knowledge, the first cases of X-linked CGD chorioretinitis in female carriers, possibly due to skewed X-inactivation (lyonization). Methods: Comprehensive multimodal imaging, including color fundus photography, short-wavelength autofluorescence, and spectral domain optical coherence tomography (OCT), was conducted. Functional assessment was completed with full-field electroretinogram (ff-ERG). Results: This report details two sisters with X-linked CGD carrier status, both presenting chorioretinal lesions on fundoscopy. Observed features included punched-out chorioretinal lesions, perivascular atrophy, and peripheral pigment changes. Autofluorescence imaging confirmed hypoautofluorescent areas correlating with chorioretinal atrophy, and OCT revealed retinal collapse and ellipsoid zone loss in one sibling. Despite these structural changes, visual function remained stable with minimal progression over time. Subsequent serial ERGs did not show progression. Conclusions: The findings highlight that skewed X-inactivation may contribute to retinal changes in asymptomatic CGD carriers, underscoring the need for awareness of potential ocular manifestations in X-linked genetic disorders in female carriers.

## 1. Introduction

Chronic granulomatous disease (CGD) is a rare primary immunodeficiency with a genetic basis, affecting approximately 1 in 200,000 live births [1]. CGD results from mutations that impair the function of the nicotinamide adenine dinucleotide phosphate (NADPH) oxidase enzyme complex, leading to an increased susceptibility to catalase-positive bacterial and fungal infections [2]. The majority of cases (90%) are due to mutations in the CYBB gene, encoding a component of the cytochrome b558 complex, or the *NCF1* gene, both of which are essential for proper immune function [1]. While CGD primarily presents with X-linked inheritance patterns, autosomal recessive forms also exist [1].

Due to the X-linked nature of the CYBB mutation, CGD is typically symptomatic in males. Female carriers generally remain asymptomatic, although recent studies have shown that a subset may exhibit reduced NADPH oxidase activity in up to 80% of phagocytes, attributed to skewed X-chromosome inactivation (lyonization). This skewing can lead to a carrier state with symptoms mimicking CGD [3,4]. Notably, while chorioretinal findings have been reported as an early manifestation in male infants with CGD [5], similar presentations in female carriers have not been described in detail.

Here, we present what we believe to be the first documented case of chorioretinal findings in female carriers of X-linked CGD prior to systemic manifestation. This case series adds insights into the ocular phenotype associated with X-linked lyonization in asymptomatic carriers, broadening our understanding of CGD’s clinical spectrum.

## 2. Case Descriptions

### 2.1. Case 1

**Figure 1:**
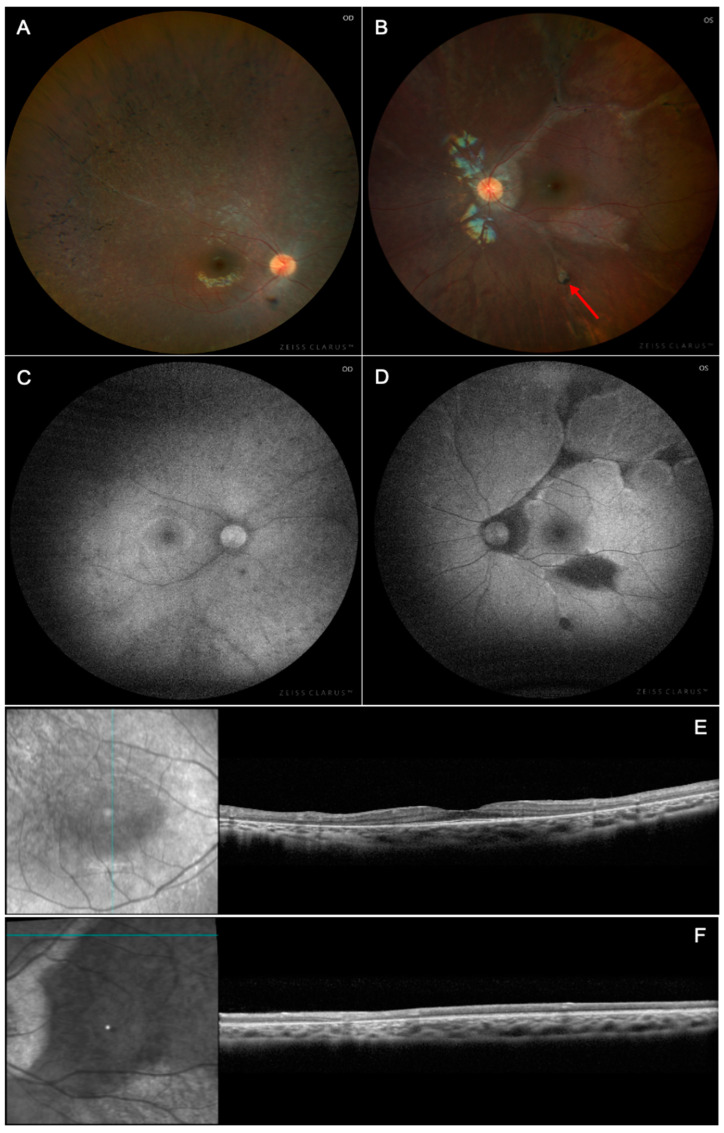
Multimodal imaging of X-linked chronic granulomatous disease chorioretinitis (Case 1). (**A**,**B**) Color fundus photographs after 2-year follow-up revealed bone spicule pigmentation in the peripheral retina bilaterally. (**B**) demonstrates extensive chorioretinal atrophy in the superotemporal arcade with punched-out chorioretinal lesions inferiorly (red arrow). (**C**,**D**) Short-wavelength autofluorescence demonstrates a hyperautofluorescent ring in the right eye and hypoautofluorescent regions corresponding to the perivascular atrophy and inferior chorioretinal lesions. Spectral domain optical coherence tomography (**E**,**F**) of the left eye demonstrated collapse of the retina perivascularly in the right eye and loss of the ellipsoid zone with foveal sparing.

At presentation, a 3-year-old girl with carrier status of X-linked chronic granulomatous disease-4 (CGD) confirmed by dihydrorhodamine testing was found to have a pale retinal area as per color fundus photograph on a routine eye evaluation. She was then referred to retina specialists who were concerned for retinitis pigmentosa, and she was referred to Columbia University for further neuro-ophthalmic evaluation. The patient had amblyopia in the right eye, and her family denied any past ocular history. On initial presentation, her best-corrected visual acuity (BCVA) was 20/30 +1 in both eyes. Her anterior segment examination was unremarkable; however, on dilated fundus examination of the right eye she was found to have arterial attenuation and bone spicule pigmentation in the peripheral retina (Figure 1A). Fundoscopic examination of the left eye demonstrated perivascular chorioretinal atrophy at the superotemporal arcade, bone spicule pigmentation and mottling in the peripheral retina, and a punched-out chorioretinal lesion inferiorly (Figure 1B). Autofluorescence of the right eye revealed a hyperautofluorescent ring surrounding the macula (Figure 1C). Autofluorescence of the left eye revealed hypoautofluorescence perivascularly at the superotemporal arcade and hypofluorescence lesions in a punched-out pattern inferiorly (Figure 1D). Notably, optical coherence tomography (OCT) demonstrated outer retinal atrophy perivascularly in the right eye and loss of the ellipsoid zone with foveal sparing in the left eye (Figure 1E,F). Toxoplasmosis, rubella, cytomegalovirus, herpes simplex, and syphilis were ruled out by blood test. ESR and CRP were within normal limits.

**Figure 2:**
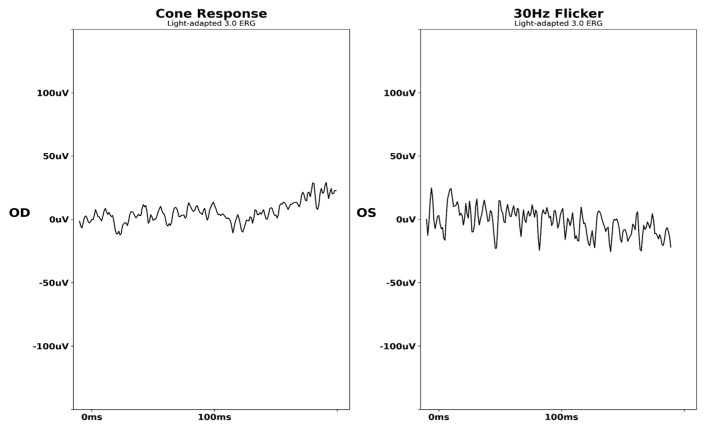
Initial full−field electroretinogram of X−linked chronic granulomatous disease chorioretinitis (Case 1). FF−ERG interpretation was limited due to blinking, eye movement, and limited patient compliance.

Given her asymmetric findings, RPGR-associated retinitis pigmentosa was suspected. Panel-based genetic testing of 248 genes associated with inherited retinal disorders was obtained through Invitae (San Francisco, CA, USA) and revealed heterozygous mutations in genes encoding *PDE6B* c.55G>T:p.(Ala19Ser) and *ZNF423* c.3280G>A:p.(Gly1094Ser), both variants of uncertain significance and clinically not deemed the cause of her retinal condition. Expanded genetic testing of 770 genes associated with inherited retinal disorders obtained through GeneDx (Gaithersburg, MD, USA) was also negative. After four years of annual monitoring, the patient received a full-field electroretinogram (ff-ERG). Interpretation was limited given patient movement, a common issue during childhood, and only the right eye was examined due to patient discomfort (Figure 2).

**Figure 3:**
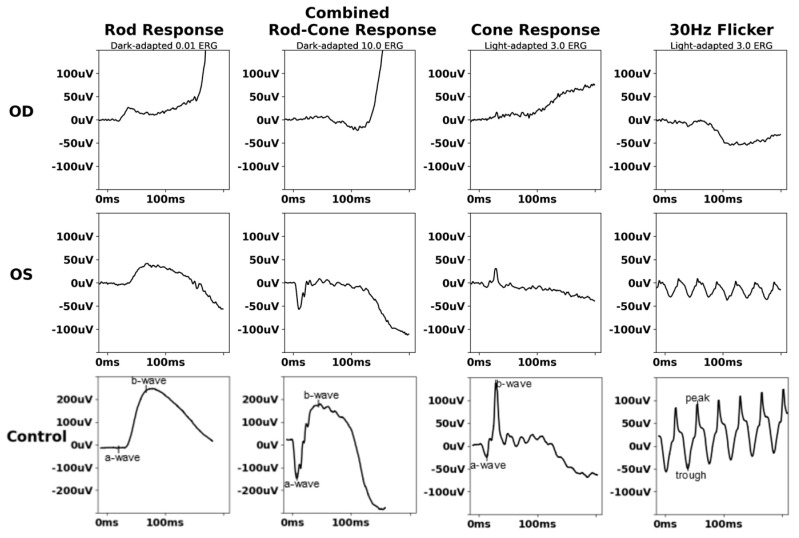
Follow−up full−field electroretinogram of X−linked chronic granulomatous disease chorioretinitis (Case 1). FF−ERG demonstrated diminished scotopic amplitudes with normal photopic response in OS.

Repeat ERG 4 years later was tolerated better and was suggestive of rod–cone dysfunction (Figure 3). Subsequent serial ERG did not show progression.

At 9 years old, she has continued to have no progression in symptoms and exam findings. Based on the clinical evaluation and laboratory results, the fundus and OCT findings are most consistent with a carrier state of CGD. X-linked inactivation due to lyonization is a plausible explanation for these observed features.

### 2.2. Case 2

**Figure 4:**
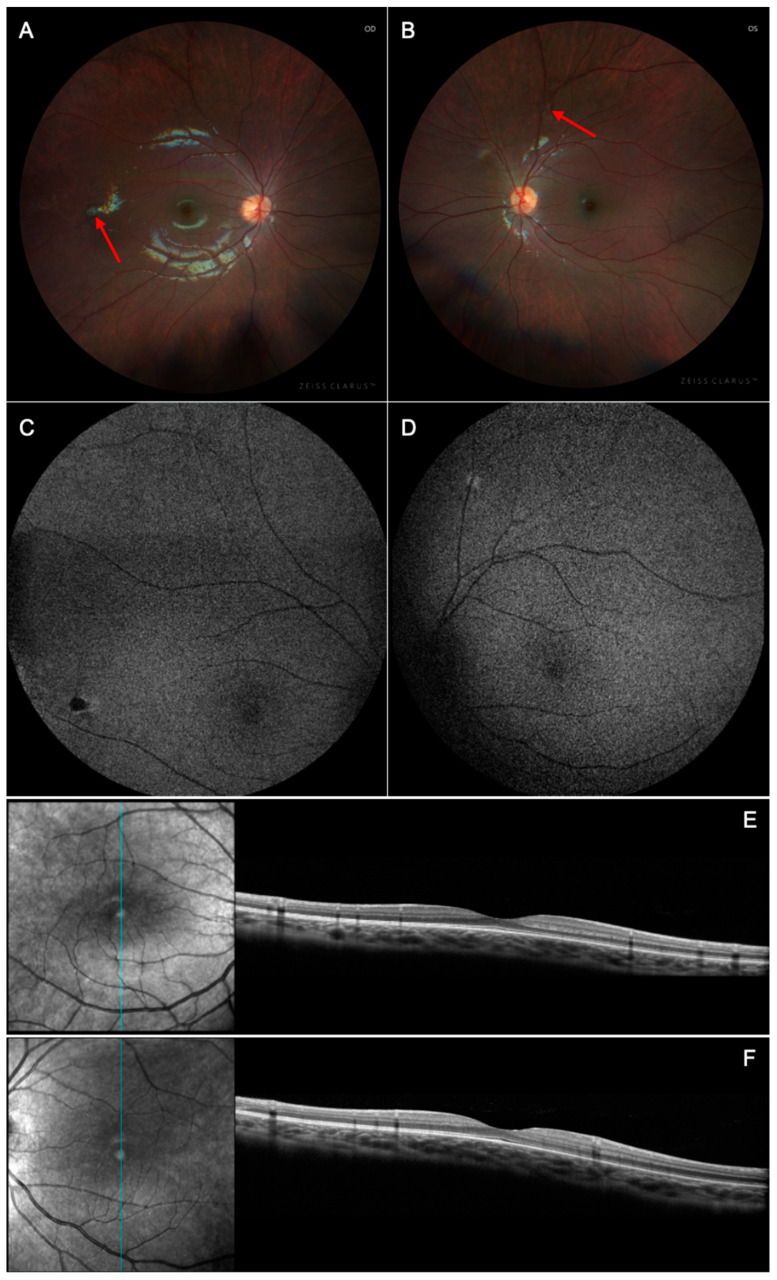
Multimodal imaging of X-linked chronic granulomatous disease chorioretinitis (Case 2). (**A**,**B**) Color fundus photographs revealed hypopigmented punched-out chorioretinal lesions bilaterally (red arrow). (**C**,**D**) Short-wavelength autofluorescence demonstrated hypoautofluorescent and hyperautofluorescent lesions corresponding to the OD and OS fundus exam findings, respectively. (**E**,**F**) Spectral domain optical coherence tomography was unremarkable.

At initial presentation, the 5-year-old younger sister of Case 1, also with carrier status of X-linked CGD confirmed by dihydrorhodamine testing, was found to have a similar pale area as per color fundus photograph on a routine eye evaluation, so she was also referred to Columbia University for neuro-ophthalmic evaluation. She had no past ocular history, and denied visual changes. She had a past medical history of Hashimoto disease controlled with levothyroxine 25 mcg daily. On initial presentation, her BCVA was 20/30 in both eyes. Her anterior segment examination was unremarkable; however, dilated fundus examination of the right eye revealed a punched-out chorioretinal lesion temporally (Figure 4A). Fundoscopic examination of the left eye revealed a hypopigmented punched out lesion superiorly (Figure 4B). Autofluorescence of the right eye demonstrated a hypoautofluorescent lesion temporally corresponding to the fundus exam finding (Figure 4C). Autofluorescence of the left eye demonstrated a hyperautofluorescent lesion superiorly corresponding to the fundus exam finding (Figure 4D). OCT was unremarkable (Figure 4E,F).

**Figure 5:**
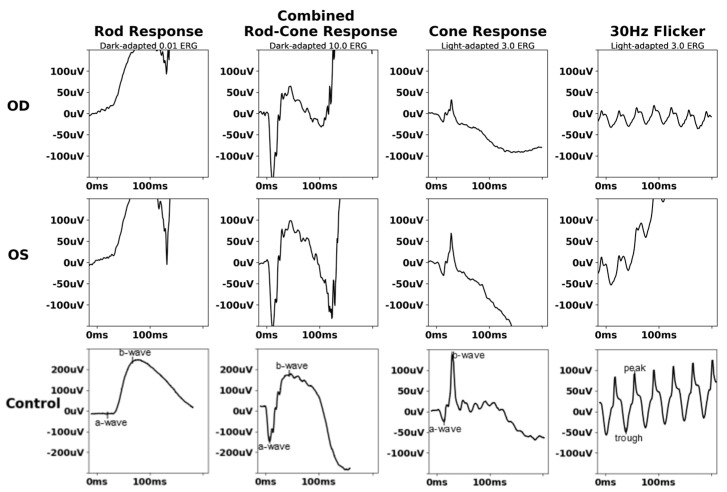
Full−field electroretinogram of X−linked chronic granulomatous disease chorioretinitis (Case 2). FF−ERG displayed normal scotopic and photopic b−wave and flicker peak times.

2 years later at a follow-up visit, dilated fundus examination revealed mottling and intraretinal pigment migration in the periphery of both eyes. Ff-ERG was performed demonstrating full scotopic and photopic function (Figure 5). Toxoplasmosis, rubella, cytomegalovirus, herpes simplex, and syphilis were ruled out by blood test. ESR and CRP were within normal limits. Table 1 summarizes both cases.

## 3. Discussion

The care team acknowledged retinitis pigmentosa as a differential diagnosis in both cases, as it is the leading cause of inherited blindness [6]. However, given unilateral clinical features, lack of progression following four years of annual follow-up, and a father with confirmed X-linked CGD due to CYBB mutation, X-linked CGD was the presumed diagnosis [1].

Chorioretinal lesions associated with CGD were first identified in 1965 in a series of male patients, who typically exhibit more severe symptoms due to their hemizygous status [7]. These lesions included RPE atrophy or pigment clumping, chorioretinal atrophy, and punched-out lesions [7]. This case series adds novel insights by documenting similar ophthalmic findings in female carriers, highlighting the impact of lyonization in X-linked diseases.

Lyonization or X-Chromosome inactivation, first described in 1961 by Mary Lyon, is the process of random inactivation in one copy of the X-chromosome for dosage compensation. Either the paternal or maternal X-chromosome is randomly selected to be inactivated, occurring in early embryogenesis and remaining inactive in all subsequent cell generations. This random deviation results in mosaic patterns in females. Consequently, lyonization in female carriers of X-linked diseases can result in variable clinical manifestations. For instance, as shown in our reports, even two siblings presumed to be carriers of CYBB mutations exhibit differing ophthalmic manifestations, potentially due to differences in the degree of lyonization [8].

In this scenario, neutrophils that remain unaffected by lyonization retain an intact respiratory burst, while those subject to X-inactivation of CYBB exhibit an impaired response, mimicking CGD [9]. This environment with impaired neutrophil activity yields a hyperinflammatory state that causes chorioretinal changes seen in both cases [1]. Goldblatt et al. reported that 9 out of 38 affected individuals with chorioretinal lesions were male. The observed fundus findings included punched-out areas of chorioretinal atrophy and pigment clumping, resembling the findings in our first case. However, in our report, another case with skewed XCI presented with a milder degree of chorioretinal atrophy [9].

Typically, female carriers of CYBB mutations are asymptomatic due to a sufficient proportion of cells expressing wild-type CYBB, resulting in an overall normal phenotype [10]. However, despite the absence of systemic symptoms, both patients demonstrated chorioretinal findings—a novel observation for previously asymptomatic X-linked CGD carriers—illustrating how lyonization can manifest ocularly in X-linked diseases. This phenomenon parallels findings in other X-linked retinal disorders, such as X-linked retinitis pigmentosa, where female carriers may develop mild to moderate retinal degeneration due to skewed X-inactivation, in comparison to males with X-linked disease. The potential for lyonization to yield phenotypic variability even in female carriers calls for increased surveillance and comprehensive evaluation in all X-linked diseases. X-linked mutations may manifest in carriers across other diseases.

The observation of chorioretinal changes in these CGD carriers before any systemic manifestations highlights the importance of early ophthalmic evaluations in female carriers of X-linked disorders. Multimodal imaging, and analyzing color fundus images, autofluorescence, and OCT progression over time, can reveal subtle ocular changes, namely chorioretinal atrophy, that may serve as early markers of carrier status and prompt further evaluation [6,11,12,13]. Furthermore, the clinical presentation in affected carrier females may vary widely. Thus, we highlight the need for functional assessment of neutrophil activity, the dihydrorhodamine (DHR) assay used in both cases for example, and detailed phenotyping in all patients with known family history of X-linked CGD. We recommend annual ERGs as a non-invasive objective tool useful for assessing retinal function and elucidating etiology of disease, as inflammatory conditions tend to not show progression [1], as shown in Case 1. Table 2 depicts the electrophysiology markers necessary for clinical evaluation in those with retinal disease [14]. In both cases, ff-ERG findings suggest absent–minimal decline, aligned with good prognosis. This case series supports a proactive approach to screening, where routine retinal imaging could facilitate early detection and improve clinical outcomes by enabling timely interventions [5].

X-linked CGD has shown to have a more severe disease course with earlier presentation and earlier age at death [15]. However, in cases where systemic infections may develop at a later age, identifying carriers through retinal signs could provide an opportunity for early detection/monitoring and prophylactic care via antibiotics, antifungals, and interferon-gamma to improve immune function. Such insights can be especially relevant in clinical practices involving genetic screening and counseling for families with known X-linked mutations.

The cases presented here reinforce the value of genetic counseling for families affected by X-linked CGD, given the potential for variable expression among carriers. Genetic counseling can provide family members with an understanding of the inheritance patterns, likelihood of carrier status, and potential ocular or systemic manifestations. Furthermore, this counseling could guide decisions regarding ophthalmic and systemic surveillance, emphasizing that female carriers may not be entirely asymptomatic and may benefit from a structured monitoring plan. By recognizing the possibility of lyonization-induced symptoms, genetic counseling can also set expectations for clinical variability and inform family planning decisions.

This study is limited by its sample size, which constrains the generalizability of findings. Further studies are needed to assess the prevalence and progression of chorioretinal changes in larger cohorts of female CGD carriers. Additionally, prospective studies exploring the long-term visual outcomes and potential interventions could inform management guidelines for carriers with ocular involvement. Investigating therapeutic options that may help stabilize or prevent retinal alterations associated with lyonization could represent an important avenue for improving quality of life in these patients. Research into other X-linked disorders with known ocular manifestations in female carriers could also provide comparative insights into lyonization’s clinical implications.

## 4. Conclusions

In conclusion, we report, to our knowledge, the first documented case of chorioretinal findings in female carriers of X-linked CGD prior to systemic manifestation. Several papers report case series of male X-linked CGD chorioretinal abnormalities. However, a female carrier presenting with chorioretinal findings has never been described before in the literature. Given that X-linked CGD can affect various systems, it is suggested that patients with known family history of X-linked CGD undergo regular ocular examinations until adulthood to identify and manage chorioretinal changes and begin prophylactic measures prior to systemic disease. The clinical presentation in affected carrier females may vary widely. Thus, we highlight the need for functional assessment of NADPH oxidase activity and detailed phenotyping in all patients. Furthermore, our findings suggest that lyonization-induced manifestations in carriers could have implications for other X-linked disorders—further emphasizing the need for comprehensive evaluation in this population and broadening the understanding of X-linked disease.

## Figures and Tables

**Table 1:** Comparative clinical and imaging findings in two female carriers of X-linked chronic granulomatous disease (CGD).

Feature	Case 1 (3-Year-Old Girl)	Case 2 (5-Year-Old Girl)
Carrier Status	X-linked CGD Carrier	X-linked CGD Carrier
Presentation	Pale area on fundoscopy	Pale area on fundoscopy
Systemic History	None Reported	Hashimoto’s disease controlled with levothyroxine
Best-Corrected Visual Acuity (BCVA)	20/30 +1 in both eyes	20/30 in both eyes
Anterior Segment Findings	Unremarkable	Unremarkable
Dilated Fundus Examination	OD: Arterial attenuation, peripheral bone spicule pigmentation OS: Perivascular chorioretinal atrophy, peripheral bone spicule pigmentation, inferior punched-out lesion	OD: Temporal punched-out lesion OS: Superior hypopigmented lesion
Autofluorescence Imaging	OD: Hyperautofluorescent ring around macula OS: Hypoautofluorescent perivascular areas and inferior punched-out lesions	OD: Temporal hypoautofluorescent lesion OS: Superior hyperautofluorescent lesion
Optical Coherence Tomography (OCT)	OD: Retinal collapse perivascularly OS: Ellipsoid zone disruption with foveal sparing	Unremarkable
Follow-up Fundus Changes	Stable chorioretinal findings over time	Peripheral mottling and intraretinal pigment migration
Full-Field Electroretinogram (ff-ERG)	Rod–cone dysfunction	Full scotopic and photopic function
Genetic Testing Results	Variants of uncertain significance (PDE6B and ZNF423); expanded panel negative Dihydrorhodamine testing confirming *CYBB* carrier status	Dihydrorhodamine testing confirming *CYBB* carrier status
Final Diagnosis	CGD carrier with chorioretinal changes likely due to lyonization	CGD carrier with chorioretinal changes likely due to lyonization

**Table 2:** Comparative FF-ERG findings.

Patient ID	Scotopic Response OD and OS B Wave (µV)	Scotopic Response Time OD and OS (ms)	Maximum Response OD and OS A Wave (µV)	Maximum Response OD and OS B Wave (µV)	Cone Response OD and OS A Wave (µV)	Cone Response OD and OS B Wave (µV)	30 Hz Photopic Flicker OD and OS (µV)	30 Hz Flicker Implicit Time OD and OS (ms)
P1	4.087/45.84	100/69	−0.537/−57.2	8.529/66.26	−0.985/−9.89	17.22/40/25	5.01/35.3	25/25
P2	142.2/163.9	98/85	−158.5/−154.3	223.1/252.8	−20.02/−30.59	52.11/99.57	39.03/68.9	25/25

## Data Availability

No new data were created or analyzed in this study.
